# Supplementary material for: Oxytocin Enhancement of Emotional Empathy: Generalization Across Cultures and Effects on Amygdala Activity
Source: Front Neurosci. 2018 Jul 31;12:512. doi: 10.3389/fnins.2018.00512 (PMC6079225; doi:10.3389/fnins.2018.00512)
Supplement: Supplementary file 1 [file Data_Sheet_1.DOCX]

**Supplemental Materials**

**Oxytocin enhancement of emotional empathy: generalization across cultures and effects on amygdala activity**

YaYuan Geng^1^, Weihua Zhao^1^, Feng Zhou^1^, Xiaole Ma^1^, Shuxia Yao^1^, Rene Hurlemann^2,3^, Benjamin Becker^1*^, Keith M. Kendrick^1*^

^1^The Clinical Hospital of Chengdu Brain Science Institute, MOE Key Laboratory for Neuroinformation, University of Electronic Science and Technology of China, China

^2^Department of Psychiatry, University of Bonn, Bonn, Germany

^3^Division of Medical Psychology, University of Bonn, Bonn, Germany

^*^Joint-corresponding authors

**Correspondence**:

Keith M. Kendrick (k.kendrick.uestc@gmail.com)

Benjamin Becker (ben_becker@gmx.de)

**Experimental Stimuli**

In a first step, the Chinese stimuli were evaluated by an independent sample of 20 male Chinese students who rated the valence (scale 1-9, 1 = very negative; 9 = very positive) and arousal (scale 1-9, 1 = very calm, 9 = very aroused) of the photo-based stimuli. As expected, positive stimuli were rated more positive than negative stimuli (M ± SD, positive 7.24 ± 0.40; negative 2.48 ± 0.70, T-test: T_46.5_ = -32.46, p <0.001, Cohen’s *d* = -8.35), whereas there were no significant differences in arousal (M ± SD, positive 5.34 ± 0.75; negative 4.92 ± 1.35, T_45.23_ =0.14, p = 0.14).

**Electrodermal activity assessment**

In Exp 2, SCR was assessed with a pair of fMRI compatible electrodes provided by Biopac (Biopac Systems, Inc., Santa Barbara, CA, USA). Electrodes were attached to the second and third digits on the left hand between the first and second phalanges, connected to Biopac amplifiers and a Biopac MP 150 A/D digitization system (digitizing signals at 1kHz;). Data was acquired using AcqKnowledge software (version 4.2, Biopac Systems Inc., www.biopac.com). Electrodes were attached to subjects’ fingers approximately 10 minutes before experiments started to achieve stable impedance. Differences between the peak and the base of the waveform within 5s after stimuli onset were calculated as the level of SCR responses, were square root transformed to normalize distribution for each subject (Schiller *et al.*, 2010) and then input forwarded to SPSS for ANOVA analysis.

Potential Confounders, Experiment 1

| Measurements | PLC | OXT | T-value | P |
| --- | --- | --- | --- | --- |
| Age(yr) | 22.60 ± 0.40 | 22.28 ± 0.43 | 0.55 | 0.58 |
| Education | 15.93 ± 0.30 | 15.76 ± 0.35 | 0.38 | 0.71 |
| Empathy Quotient(EQ) | 39.73 ± 1.45 | 36.41 ± 1.78 | 1.45 | 0.15 |
| Liebowitz Social Anxiety Scale(LSAS)_Fear | 25.37 ± 1.83 | 27.72 ± 2.31 | -0.80 | 0.43 |
| Liebowitz Social Anxiety Scale(LSAS)_Avoidance | 25.20 ± 1.90 | 28.52 ± 2.36 | -1.10 | 0.28 |
| Beck Depression Inventory(BDI) | 10.50 ± 1.08 | 8.28 ± 0.92 | 1.57 | 0.12 |
| State and Trait Anxiety Inventory(STAI)_State | 41.07 ± 1.34 | 40.79 ± 1.88 | 0.12 | 0.90 |
| State and Trait Anxiety Inventory(STAI)_Trait | 41.73 ± 1.36 | 40.54 ± 1.70 | 0.55 | 0.58 |
| Positive and Negative Affect Scale(PANAS)_Positive | 29.13 ± 0.91 | 29.10 ± 1.20 | 0.02 | 0.98 |
| Positive and Negative Affect Scale(PANAS)_Negative | 15.93± 0.67 | 18.41 ± 1.32 | -1.70 | 0.095 |
| The Adult Autism Spectrum Quotient(ASQ)_Social_Skill | 4.11 ± 0.49 | 3.64 ± 0.86 | 0.47 | 0.64 |
| The Adult Autism Spectrum Quotient(ASQ)_Attention_Switch | 5.05 ± 0.39 | 4.71 ± 0.49 | 0.55 | 0.59 |
| The Adult Autism Spectrum Quotient(ASQ)_Attention_To_Detail | 4.95 ± 0.53 | 4.86 ± 0.65 | 0.11 | 0.92 |
| The Adult Autism Spectrum Quotient(ASQ)_Communication | 2.37 ± 0.35 | 2.93 ± 0.64 | -0.82 | 0.42 |
| The Adult Autism Spectrum Quotient(ASQ)_Imagination | 3.47 ± 0.28 | 3.29 ± 0.43 | 0.39 | 0.70 |
| The Adult Autism Spectrum Quotient(ASQ) | 19.95 ± 1.03 | 19.43 ± 1.66 | 0.28 | 0.78 |
| Wong Law Emotional Intelligence Scale-Chinese(WLEIS-C) | 67.20 ± 2.18 | 72.72 ± 2.49 | -1.67 | 0.10 |

**Table S1**. Demographics of ages and questionnaire scores for study subjects in Exp1 (mean ± SEM).

Potential Confounders, Experiment 2

| Measurements | PLC | OXT | T-value | P |
| --- | --- | --- | --- | --- |
| Education | 15.88 ± 0.29 | 16.08 ± 0.29 | -0.52 | 0.61 |
| Age | 21.79 ± 0.35 | 22.06 ± 0.38 | -0.45 | 0.65 |
| Empathy Quotient(EQ) | 37.97 ± 1.79 | 37.47 ± 1.36 | 0.22 | 0.82 |
| The Adult Autism Spectrum Quotient(ASQ) | 20.79 ± 1.00 | 19.94 ± 1.05 | 0.58 | 0.56 |
| The Adult Autism Spectrum Quotient(ASQ)_Social_Skill | 3.97 ± 0.45 | 3.86 ± 0.36 | 0.19 | 0.85 |
| The Adult Autism Spectrum Quotient(ASQ)_Attention_Switch | 4.82 ± 0.28 | 4.97 ± 0.27 | -0.39 | 0.70 |
| The Adult Autism Spectrum Quotient(ASQ)_Attention_To_Detail | 5.73 ± 0.44 | 4.67 ± 0.38 | 1.82 | 0.073 |
| The Adult Autism Spectrum Quotient(ASQ)_Communication | 2.42 ± 0.33 | 2.89 ± 0.40 | -0.88 | 0.38 |
| The Adult Autism Spectrum Quotient(ASQ)_Imagination | 3.85 ± 0.28 | 3.56 ± 0.27 | 0.75 | 0.46 |
| Beck Depression Inventory(BDI) | 7.61 ± 1.08 | 6.61 ± 1.23 | 0.60 | 0.55 |
| Liebowitz Social Anxiety Scale(LSAS)_Fear | 43.70 ± 1.80 | 44.22 ± 1.72 | -0.21 | 0.83 |
| Liebowitz Social Anxiety Scale(LSAS)_Avoidance | 42.27 ± 1.95 | 40.78 ± 1.89 | 0.55 | 0.58 |
| Positive and Negative Affect Scale(PANAS)_Positive | 30.06 ± 1.04 | 29.25 ± 0.90 | 0.59 | 0.56 |
| Positive and Negative Affect Scale(PANAS)_Negative | 20.09 ± 1.22 | 18.94 ± 0.90 | 0.76 | 0.45 |
| Self Esteem Scale(SES) | 31.70 ± 0.66 | 32.14 ± 0.68 | -0.46 | 0.64 |
| State and Trait Anxiety Inventory(STAI)_State | 39.67 ± 1.65 | 38.33 ± 1.09 | 0.68 | 0.50 |
| State and Trait Anxiety Inventory(STAI)_Trait | 40.55 ± 1.29 | 41.36 ± 1.28 | -0.45 | 0.66 |
| Wong Law Emotional Intelligence Scale-Chinese(WLEIS-C) | 83.39 ± 1.85 | 83.31 ± 2.25 | 0.03 | 0.98 |

**Table S2**. Demographics of ages and questionnaire scores for study subjects in Exp2 (mean ± SEM).

| Measurements | Female vs Male | | | |
| --- | --- | --- | --- | --- |
|  | PLC | | OXT | |
|  | F-value | P | F-value | P |
| Empathy Quotient(EQ) | 1.44 | 0.24 | 0.58 | 0.45 |
| The Adult Autism Spectrum Quotient(ASQ) | 2.33 | 0.13 | 0.45 | 0.51 |
| The Adult Autism Spectrum Quotient(ASQ)_Social_Skill | 2.00 | 0.16 | 0.26 | 0.61 |
| The Adult Autism Spectrum Quotient(ASQ)_Attention_Switch | 0.29 | 0.59 | 0.009 | 0.93 |
| The Adult Autism Spectrum Quotient(ASQ)_Attention_To_Detail | 1.08 | 0.30 | 0.002 | 0.96 |
| The Adult Autism Spectrum Quotient(ASQ)_Communication | 1.63 | 0.21 | 0.000 | 0.99 |
| The Adult Autism Spectrum Quotient(ASQ)_Imagination | 0.73 | 0.40 | 3.13 | 0.082 |

**Table S3**. Significance of gender differences in each treatment group for empathy and autism trait scores.

**Reference**

Schiller, D., Monfils, M.H., Raio, C.M., Johnson, D.C., LeDoux, J.E. & Phelps, E.A. (2010): Preventing the return of fear in humans using reconsolidation update mechanisms. *Nature*, **463**, 49-U51.
